# Confounding factors affecting faecal egg count reduction as a measure of anthelmintic efficacy

**DOI:** 10.1051/parasite/2022017

**Published:** 2022-04-07

**Authors:** Eric R. Morgan, Carlos Lanusse, Laura Rinaldi, Johannes Charlier, Jozef Vercruysse

**Affiliations:** 1 School of Biological Sciences, Queen’s University Belfast 19, Chlorine Gardens BT9 5DL Belfast United Kingdom; 2 Laboratorio de Farmacología, Centro de Investigación Veterinaria de Tandil (CIVETAN) (UNCPBA-CICPBA-CONICET), Facultad de Ciencias Veterinarias, UNCPBA 7000 Tandil Argentina; 3 Department of Veterinary Medicine and Animal Production, University of Naples Federico II Via Delpino, 1 80137 Naples Italy; 4 Kreavet Hendrik Mertensstraat 17 9150 Kruibeke Belgium; 5 Faculty of Veterinary Medicine, University of Gent Salisburylaan 133 9820 Merelbeke Belgium

**Keywords:** Helminths, Anthelmintic resistance, Faecal egg count reduction test, Drug pharmacology related therapeutic failures, Pharmacokinetics, Epidemiology, Effectiveness

## Abstract

Increasing anthelmintic resistance (AR) in livestock has stimulated growing efforts to monitor anthelmintic effectiveness (AE) on livestock farms. On-farm assessment of AE relies on measuring the reduction in faecal egg count (FEC) following treatment; and if conducted rigorously, qualifies as a formal FEC reduction test (FECRT) for AR. Substantial research effort has been devoted to designing robust protocols for the FECRT and its statistical interpretation; however, a wide range of factors other than AR can affect FEC reduction on farms. These are not always possible to control, and can affect the outcome and repeatability of AE measurements and confound the on-farm classification of AR using FECRT. This review considers confounders of FEC reduction, focusing on gastrointestinal nematodes of ruminants, including host and parasite physiology and demography; pharmacokinetic variation between drugs, parasites and hosts; and technical performance. Drug formulation and delivery, host condition and diet, and seasonal variation in parasite species composition, can all affect AE and hence observed FEC reduction. Causes of variation in FEC reduction should be attenuated, but this is not always possible. Regular monitoring of AE can indicate a need to improve anthelmintic administration practices, and detect AR early in its progression. Careful interpretation of FEC reduction, however, taking into account possible confounders, is essential before attributing reduced FEC reduction to AR. Understanding of confounders of FEC reduction will complement advances in FECRT design and interpretation to provide measures of anthelmintic efficacy that are both rigorous and accessible.

## Abstract (fr)

L’augmentation de la résistance aux anthelminthiques (RA) chez le bétail a stimulé des efforts croissants pour mesurer l’efficacité des anthelminthiques (EA) dans les élevages. L’évaluation à la ferme de l’EA repose sur la mesure de la réduction du nombre d’œufs dans les selles (NOS) après le traitement ; si effectué rigoureusement, celui-ci est qualifié de test formel de réduction du NOS (TFRNOS) pour la RA. Des efforts de recherche substantiels ont été consacrés à la conception de protocoles robustes pour le TFRNOS et son interprétation statistique, mais un large éventail de facteurs autres que la RA peuvent affecter la réduction du NOS dans les exploitations. Ces facteurs ne sont pas toujours contrôlables et peuvent affecter le résultat et la répétabilité des mesures de l’EA et introduire de la confusion dans la classification à la ferme de la RA à l’aide de la TFRNOS. Cette revue examine les facteurs de confusion de la réduction du NOS, en se concentrant sur les nématodes gastro-intestinaux des ruminants, et considère la physiologie et la démographie de l’hôte et du parasite, la variation pharmacocinétique entre les médicaments, les parasites et les hôtes, et les performances techniques. La formulation et l’administration de médicaments, la condition et le régime alimentaire de l’hôte, ainsi que les variations saisonnières de la composition des espèces de parasites, peuvent tous affecter l’EA et donc la réduction du NOS observé. Les causes de variation de la réduction du NOS doivent être atténuées, mais cela n’est pas toujours possible. Une surveillance régulière de l’EA peut indiquer la nécessité d’améliorer les pratiques d’administration des anthelminthiques et de détecter la RA au début de sa progression. Une interprétation prudente de la réduction du NOS, cependant, en tenant compte des facteurs de confusion possibles, est essentielle avant d’attribuer la réduction du NOS à la RA. La compréhension des facteurs de confusion d’une moindre réduction du NOS complétera les progrès sur la conception et l’interprétation du TFRNOS, pour fournir des mesures de l’efficacité des anthelminthiques à la fois rigoureuses et accessibles.

## Introduction: measuring anthelmintic efficacy using faecal egg counts

The intensive use of anthelmintic drugs to control parasites in livestock has led to high levels of anthelmintic resistance (AR) worldwide, especially in grazing ruminants and horses [37, 65, 78, 88], and associated negative economic impacts [12, 67]. Consequently, the prevention, diagnosis and management of AR are dominant research priorities in veterinary helminthology [13, 69]. The faecal egg count reduction test (FECRT) is the method of choice for diagnosing AR in the field, being the only method that works widely across drug classes and parasite species, at least within the gastrointestinal nematodes (GIN) [37]. The principle of the FECRT is simple: egg density in host faeces (= faecal egg count, FEC) is enumerated at the time of treatment and at a defined time following treatment, and the reduction in FEC is used to indicate drug efficacy. An acceptable limit is set on proportional reduction of FEC, typically 95%, and inferior FEC reductions (FECR < 95%) lead to a classification of AR, provided the FECRT has been conducted correctly.

Methods for conducting and interpreting FECRT for nematodes of veterinary importance are set out by the World Association for the Advancement of Veterinary Parasitology (WAAVP) ([16]; update due 2022). Recommended methods make some pragmatic compromises: notably, in defining 95% as the critical threshold for FECR, and in specifying various post-treatment sampling intervals between 7 and 17 days for different drug groups. The re-sampling interval for GIN trades the potential for maturation of surviving immature worms and/or re-infection, hence egg production by worms not present as adults at the time of treatment, against the possibility of temporary suppression of fecundity in worms surviving treatment [61].

## Anthelmintic effectiveness versus efficacy

Substantial research has characterised factors affecting the design and interpretation of the FECRT, mainly focusing on increasing confidence in a classification of AR (FECR < 95%) [8, 23, 53, 54, 99]. Higher FEC sensitivity, a larger number of individual animals sampled, and more sophisticated statistical analysis, increase confidence in the FECRT result, against a backdrop of natural variation in individual parasite burdens and egg distribution in faeces. Many factors other than AR, however, can cause smaller-than-expected reductions in FEC following treatment. Thus, anthelmintic efficacy is defined as ability to clear worm infections under ideal conditions and presupposes a healthy animal and delivery of the desired drug dose to the parasites [101]. Failure of treatment to clear worms under these conditions is a strong indicator of AR. If treatment fails for reasons other than heritable anthelmintic resistance in the parasite population, however, anthelmintic effectiveness can be said to be reduced from expected levels, but AR cannot be inferred. Consequently, in discussing confounders of the FECRT, we here choose to make the critical distinction between efficacy, i.e., effect under ideal conditions and the inverse of AR; and effectiveness, i.e., effect in the real world.

It is very important to distinguish between reduced anthelmintic effectiveness (AE), also known as therapeutic failure, and AR (reduced efficacy due to heritable resistance), since the implications for continued use of a given class of anthelmintic differ radically. Hence, in the case of AR, heritable resistance is present and likely to worsen if management is not changed, and recommendations could include switching to a different drug and altering treatment strategies to alleviate selection pressure [11]; whereas poor effectiveness in the absence of AR should prompt a review of treatment procedures. In practice, it can be difficult to make this distinction, and declining AE should prompt caution around anthelmintic treatment strategies, and confirmatory FECRT (Table 1).

**Table 1:** Recommendations for faecal egg count reduction (FECR)-based testing of anthelmintic effectiveness (AE) in gastrointestinal nematodes of livestock, and inference of anthelmintic resistance (AR) using the FEC reduction test (FECRT), in the light of confounders on farms.

Action	Comment
Understand AE versus AR	AR is reduced efficacy, and hence effectiveness, that arises from heritable resistance to the drug; whereas reduced effectiveness can occur in susceptible populations due to other factors. Confusing poor AE (= therapeutic failure) with AR is misleading.
Exclude confounders	Make every reasonable attempt to exclude confounders of FECR, by ensuring accurate drug delivery, to animals without issues likely to interfere with bioavailability, carrying representative parasite burdens, and using high technical standards at treatment, sampling and laboratory stages.
Document residual confounders	Note those confounders that cannot be excluded and consider them in the interpretation of FECR results.
Heed level of effectiveness	AR misclassification risk is most affected by FEC variation and confounders when FECR is close to 95%, but is lower further from this threshold; the level of FECR, and not only binary classification as resistant or susceptible (or, for AE, effective or not), is relevant to farm management decisions.
Repeat measurements	Do not rely on a single FECRT to definitively diagnose the AR status of a herd or farm, since this can be misleading; results will vary according to time and other factors, so repeat the test when possible. Track AE alongside routine farm management and follow up low FECR with further investigations.
Identify parasite species	Parasite species information before and after treatment, if available, will improve FECR reliability and enable targeted remedial measures in response to poor AE or AR.
Communicate limitations	There are fundamental reasons for variation in FECR results. Denying them could fuel false expectations and devalue the test as the best available indicator of AR in most situations. Make management decisions in light of %FECR and uncertainty around the test result and not just apparent AR classification.
Nudge behaviour change	Use FECR and other data as an opportunity to stimulate and engage with holistic approaches to parasite management, to preserve remaining anthelmintic efficacy.

## Monitoring anthelmintic effect in the real world

Monitoring of AE on farms is promoted as a way to identify drug failure at an early stage in the development of AR, when management can be altered in time to prolong the useful life of anthelmintics at farm and regional scales. Such monitoring often makes use of opportunities to assess AE alongside routine farm management activities, for example anthelmintic treatments to support productivity, which can introduce additional variability. Effectiveness and resistance are often conflated in practice, because monitoring of AE and classification of AR both use FECR as the key metric, and because the purpose of AE monitoring is usually to identify therapeutic failure as a result of AR. Although FECR < 95% does not necessarily denote AR, it is often taken to indicate that a given anthelmintic is failing due to parasite resistance, and to provoke a review of parasite control practices. Alternatively, AR might be present and yet the FECRT returns FECR > 95%, conferring a false sense of security and a missed opportunity for constructive management change. Simplifications of the FECRT, such as using pooled samples [31, 76] or dispensing with the control group [37], are designed to improve practicality and uptake on commercial farms, but tend to decrease confidence in the result. Alongside factors that reduce AE in the absence of AR, the methodology and technical rigour used when conducting FECRT under commercial farm conditions often deviate unavoidably from ideal conditions, and lead to erroneous classification of both AE and AR.

**Figure 1:**
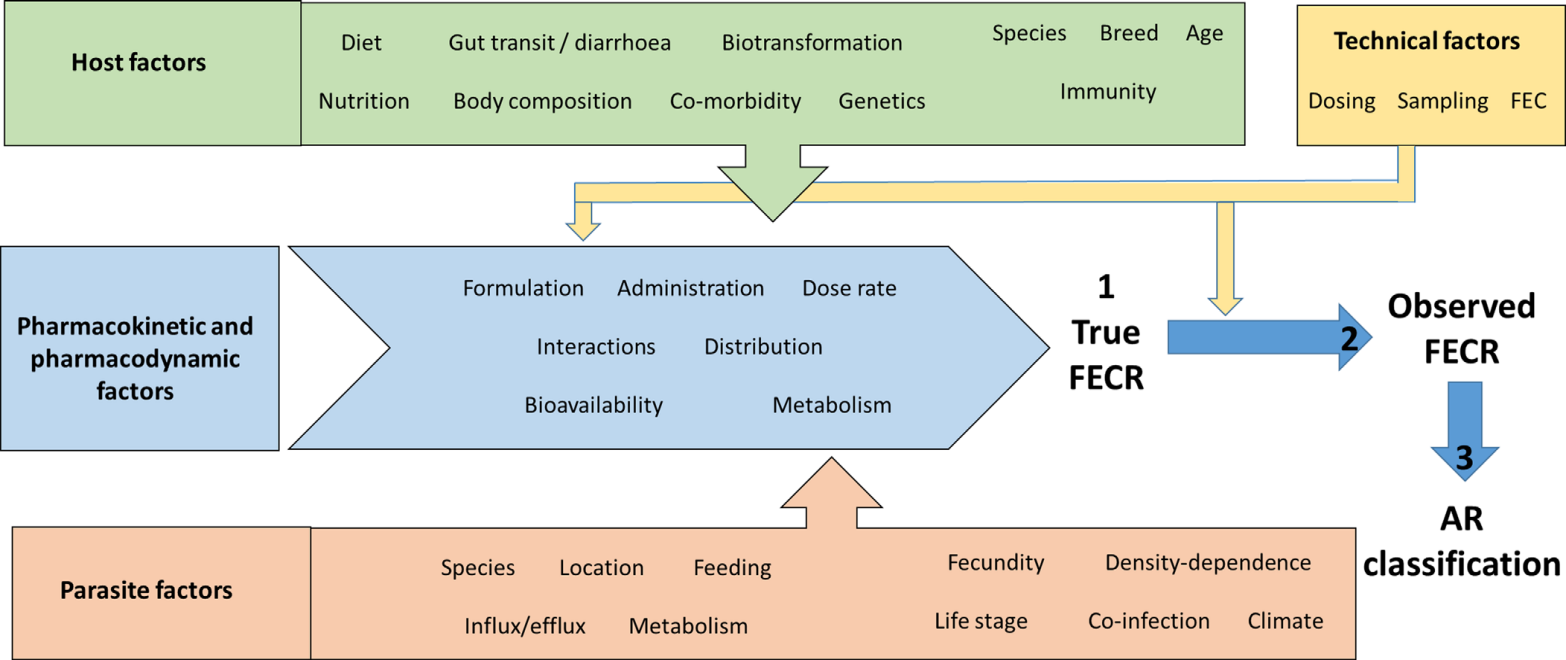
Schematic showing the range of confounders potentially influencing faecal egg count reduction (FECR) following anthelmintic treatment, and hence classification of anthelmintic resistance (AR). These are divided into host, parasite and technical factors, which together affect actual reduction in faecal egg count (1). Technical considerations also influence the accuracy with which FECR is observed (2), and hence the detection of anthelmintic resistance (3). Technical refinements to the FECRT have very much focused on improving the accuracy with which actual FECR is measured and translated into AR classification (step 3), even though many factors other than AR can strongly influence actual FECR. These risk confounding the FECRT and should be borne in mind when designing, conducting and interpreting the test, whether in standardised form for detection of AR, or in modified forms to monitor anthelmintic effectiveness.

While advances in statistical analysis and improved FEC methods decrease the risk of AR misclassification due to technical error, biological processes can also confound FECR (Fig. 1) and lead to erroneous conclusions on AE and AR. Focusing on GIN in ruminants, this review identifies and discusses these confounding factors, awareness of which can help to avoid pitfalls in interpretation and stimulate research to meet practical needs in the field. Pharmacological and pharmacokinetic factors are discussed first, followed by those arising from variation in host and parasite populations, and then the measurement of FECR.

## Pharmaco-therapeutic confounders

The effectiveness of anthelmintics depends on exposing the parasite to an adequate drug concentration for sufficient time to achieve the target efficacy. If this is not achieved, effectiveness will be low even in the absence of AR. To guard against misinterpretation of poor AE, therefore, it is important to understand the pharmacology of the main anthelmintic chemical families, which depend on the pharmacokinetic and pharmacodynamic mechanisms of drug delivery and action [46, 55]. Pharmacokinetics involve the time course of drug absorption, distribution, metabolism and elimination from the host, which, in turn, determine the concentration of the active drug reaching both the parasite location and the target worm. Understanding the complex interactions among drug physicochemical properties, pharmaceutical preparations, routes of administration and dose rate, which directly influence the resultant kinetic behaviour and therapeutic efficacy, is critical to maximize drug effect [45], and to identify potential confounding issues on FECR outcomes. There is a strong relationship between pharmacokinetics and pharmacodynamics (drug effect), and the pharmacokinetics of anthelmintic drugs differ greatly between the different chemical groups [55].

Considering the complex factors that can affect parasite exposure to effective concentrations of anthelmintic, it follows that any adverse pharmacokinetic issue can decrease AE and cause therapeutic failure, and if not accounted for can lead to misclassification of AR using the FECRT.

### Bioavailability and administration route

It is often assumed that, in the gut, it is the intraluminal concentration of anthelmintic that is critical for parasite exposure. However, anthelmintics are absorbed and metabolized in a variable fashion, and drug metabolites may or may not be active against the parasite. Some anthelmintics, notably many benzimidazoles (BZD), are not active in the form administered and require biotransformation, for example in the liver, gut and in other tissues, to then reach the target parasites. GIN may be exposed to the active form of anthelmintics while feeding on blood, tissue fluid or gut secretions, or by recirculation of the compound or active metabolites through the enterohepatic circulation [45]. Transcuticular diffusion is also an important drug intake route for non-suckling GIN [45].

Dissolution of drug particles in gastrointestinal fluids is particularly important for drugs administered as suspensions by the oral route (such as BZD, morantel/pyrantel, etc.). On the other hand, anthelmintics parenterally injected as drug solutions, including some formulations of macrocyclic lactones (ML) or levamisole (LEV), do not require dissolution before systemic absorption, and the digestive secretion process is an important step to assure drug-nematode contact in the gut. Drug absorption is a main limiting factor that determines the amount of drug reaching the systemic circulation. The reversible exchange between the bloodstream and tissues then allows the drug and/or its metabolites to achieve concentrations that are anthelmintically active in the tissues occupied by parasites [3].

Some orally administered anthelmintics, notably the benzimidazole (BZD) methylcarbamates (albendazole, fenbendazole, etc.) have limited water solubility and small differences in drug solubility may have a major influence on their absorption, systemic availability and resultant clinical efficacy [47]. While strategies to optimise solubility have been investigated, poor or erratic gastrointestinal absorption is common for orally administered BZD suspensions in ruminant species [46], and consequent therapeutic failures should be seriously considered when results from FECRT are being analysed.

Macrocyclic lactones (ML) can be administered by oral or parenteral routes, or trans-cutaneously via pour-on formulations. Oral administration of ML facilitates the achievement of higher drug concentrations and enhanced efficacy against nematodes located in the gastrointestinal tract compared to transcutaneous treatment in sheep, cattle and horses [49, 58, 85]. This difference in activity is magnified if the parasite population has reduced susceptibility [32, 58].

Following administration of ML pour-on formulations to cattle, anthelmintic exchange between cattle through natural grooming behavior, i.e., self- or allo-licking, can significantly impact on AE [6]. Higher and more variable systemic availability of ivermectin [44] and doramectin [80] were observed in licker cattle, compared with animals whose licking behaviour was prevented. The inter- and intra-animal variability associated with licking behaviour should be considered a biological fact influenced by social, nutritional, physiological, pathological, environmental, and management factors. It has consequently been recommended that the pharmaco-parasitological evaluation for regulatory approval of pour-on products in cattle be conducted when animals are not prevented from allo- and self-licking [89], and allowing this behaviour is presumably also important in assessment of AE on farms.

Human factors can cause variation in the dose of anthelmintic administered, for example through poor calibration of dosing equipment, inaccurate estimation of animal weight, or failure to part the coat sufficiently well to ensure contact of topically applied anthelmintic with the skin. Once applied, absorption of topical anthelmintic can be affected by other factors, such as bathing or heavy rainfall – although ivermectin pour-on efficacy against *Cooperia* in cattle was not found to be affected by rainfall [75]. Given that untreated animals can ingest topical anthelmintic from others, some drug effect might occur in the control group of FECRT using these formulations, leading to false positives for AR. Since animal behaviour can affect bioavailability of pour-on anthelmintics, it is recommended that evaluation of AR on farms use oral or injectable formulations.

### Formulation quality and drug combinations

Differences between batches in the quantity of the active ingredient, its bioavailability, or degradation during storage and transport may result in variable efficacy. For luminal-acting anthelmintics, particle size is very important, with fine particulate size far superior to coarse; this is affected further by passage along the intestine [39]. Erratic absorption and variable systemic availability can be expected after administration of low quality BZD suspensions, where large drug particle size and poor pharmaco-technical elaboration may affect the rate of dissolution and resultant absorption in the gastro-intestinal (GI) tract [100]. These issues affect AE in ruminants. Hence, marked differences in systemic exposure and efficacy were observed among different commercially available generic albendazole [87] and triclabendazole [71] formulations in sheep, associated with the impact of pharmaceutical quality on the dissolution of drug particles and resultant GI absorption. Additionally, major differences in drug kinetic behaviour were observed for different commercially available ivermectin formulations in cattle [56, 57]. Anthelmintics must meet the International Pharmacopoeia standard of dissolution and disintegration times that may affect drug efficacy [1]. Products failing to meet quality standards could lead to therapeutic failure. While it is obvious that evaluation of AR using FECRT should only use high quality anthelmintic formulations that have been properly stored, practical monitoring of AE relies on commercially available preparations and increasingly on treatments given as part of routine farm management. These are selected by farmers according to their preferences, including convenience and cost. Furthermore, in markets where poor quality formulations, or even counterfeit drugs [40], are common, it can be very difficult to exclude the confounding effect of sub-optimal formulation.

Combinations of two or more anthelmintics are increasingly used to manage AR [46]. The occurrence of potential pharmacokinetic and/or pharmacodynamic interactions between drug components, however, could alter their effectiveness [45]. For instance, the residence time of oxfendazole and triclabendazole in sheep was affected by co-administration [98]. As multi-drug resistance becomes more common, evaluation of multiple co-administered actives, whether in combined formulations or sequentially, is increasingly relevant, and could be affected by such pharmacokinetic interactions.

Pharmacokinetic and pharmacodynamic confounders of AE are carefully excluded from therapeutic trials needed for drug registration [101], with good reason, but are common on farms. To recommend that they also be excluded from FECRT is sensible but not always practical, especially when combined with host factors.

## Host confounders

Many host-related factors may affect the kinetic behaviour and resultant clinical efficacy of anthelmintic compounds, through the processes of dissolution, absorption, and biotransformation.

### Species, breed and individual differences in drug metabolism

Differences in anthelmintic metabolism occur between host species and also within them according to breed and age. Pronounced pharmacokinetic differences among animal species have been documented for different anthelmintic molecules, with lower systemic availability in goats compared to sheep treated at the same dose rates, being among the most established. The rate of liver cytosolic production of reduced flubendazole metabolites can differ nearly 100-fold between sheep and pigs [64]. Within species, slow moxidectin absorption, delayed peak plasma concentration and decreased systemic exposure were observed in Aberdeen Angus compared to Holstein calves after topical treatment [79]. Plasma concentrations of ivermectin also differed between Belgian Blue and Holstein calves following subcutaneous injection [93]. Expected efficacy (effectiveness under ideal conditions) therefore differs between breeds, and while differences in bioavailability might have negligible impact on FECR when efficacy is high, the early detection of AR at marginal efficacy reduction could be affected by breed, age and related factors.

In young ruminants, orally administered anthelmintics may partially bypass the rumen and reticulum to enter the omasum and abomasum following closure of the oesophageal-reticular groove, a reflex especially developed in the nursing ruminant but inconsistently active in the adult [74]. Thus, variable portions of a drug solution or suspension administered orally may became divided between the rumen and abomasum, resulting in a complex absorption process and unpredictable drug efficacy, especially for those BZD anthelmintics with a low solubility in abomasal fluid such as fenbendazole and albendazole [47]. Oesophageal groove closure was stimulated in sheep by co-administration of glucose and led to decreased bioavailability and efficacy of oxfendazole against benzimidazole-resistant *Haemonchus contortus* [74], although the implications for AE of variation in diet, age and host species acting upon rumen bypass require further investigation.

Individual variation in drug pharmacokinetics will further generate inter-individual differences in therapeutic effectiveness [48], adding variation to FECR results. There is increasing recognition that dose-exposure-response relationships to many veterinary medicines vary greatly within animal populations [63]. This variation can be due to genetic differences in drug handling, age-related changes in drug distribution and metabolism, drug interactions due to concomitant therapy (e.g., with anti-inflammatory drugs or antibiotics) and co-morbidities (e.g., gastrointestinal diseases, malnutrition and immunodeficiency), all of which could affect AE. The potential role of pharmacokinetics and animal factors affecting optimal drug systemic exposure (bioavailability) should therefore be considered in cases of therapeutic failure, prior to concluding resistance.

### Diet and nutrition

Temporary feed restriction prior to anthelmintic treatment has been shown to increase the absorption and bioavailability of BZD in ruminants. Thus, fasting cattle prior to intra-ruminal albendazole treatment modified the absorption and disposition kinetics of albendazole metabolites, increasing plasma and tissue availability [83]. Starvation reduces digesta flow rates and the slower passage of anthelmintic enhances albendazole absorption. Fasting might consequently help to restore the action of anthelmintics whose potency has been compromised by resistance, as for oxfendazole in sheep [2]. Information on feed intake at time of treatment is therefore relevant to expected efficacy, and should be considered in FECR interpretation for AE and AR.

Feed type also influences gut transit and drug absorption. Binding of BZD compounds to dietary fibre can substantially modify the duration of the so-called rumen reservoir effect [33], altering the overall bioavailability of BZD and their metabolites in the bloodstream. Delayed gastrointestinal transit time and lower abomasal pH in calves fed on a concentrate-based diet, compared to those grazing on pasture, facilitated the dissolution and absorption of albendazole administered intra-ruminally as a drug suspension in cattle [82]. Diet also affects ruminal pH and modifies the microflora-mediated metabolic sulpho-reduction of BZD derivatives [96], which may further impact on the disposition kinetics and/or efficacy of these compounds in ruminants. Bioavailability of orally administered ML is also affected by diet: feeding pellets of the tannin-rich plant sainfoin to sheep resulted in lower plasma concentrations and lower effectiveness of ivermectin on GIN than control animals fed lucerne pellets [30]. As the use of bioactive plants or “nutraceuticals” increases in livestock to reduce reliance on anthelmintics [35], they might more commonly confound FECR.

GIN infection, and a change to a high-grass diet, can both reduce gut transit time and cause diarrhoea in grazing ruminants, especially at times when they are most likely to be treated under farm conditions. Under these circumstances, AE could be compromised regardless of AR. Moreover, high water content dilutes egg density in faeces and decreases observed FEC [50, 90]. FEC used for FECRT should therefore be adjusted for faecal water content, if this changes between sampling points. Treating animals with fast gut transit and watery faeces, which resolve before post-treatment sampling, could lead to low FECR through a double effect of low bioavailability and diluted pre-treatment FEC, causing false-positive FECRT results.

Prolonged feed restriction leading to poor nutritional status can negatively affect anthelmintic bioavailability through reduced BZD biotransformation in the liver and resultant pharmacokinetics [81], while body fat composition exerts a major influence on the distribution, metabolism and persistence of ML [60]. Thus, plasma concentrations of moxidectin were higher and persisted for longer in fat than in thin pigs [19]. Variable body composition could therefore explain differences in pharmacokinetic profiles with age, sex and body condition of livestock. Individual ruminants in poorer condition, including those carrying high GIN burdens, might experience different treatment outcomes even if dosed accurately to body weight.

### Infection and immunity

Parasitic infection can itself induce important changes to the pharmacokinetics, side-effects and effectiveness of anthelmintics. For example, parasite-mediated inflammatory reactions in the gut change mucosal permeability, luminal pH and gut transit time, altering BZD bioavailability. Elevation of abomasal pH, a characteristic outcome of abomasal GIN infection [10], decreases the plasma-abomasum pH gradient and reduces ionic-trapping of BZD molecules in the abomasum [4, 21]. The bioavailability of ML is also affected by parasitism, with close to 46% lower systemic exposure of moxidectin in GIN-infected lambs relative to uninfected controls following oral administration, and 54% after subcutaneous injection [52]. Doramectin was found to persist for a longer duration in lambs previously cleared of natural GIN infections [72]. While the treatment trials required for anthelmintic registration demonstrate, *ipso facto*, effectiveness in the presence of infection, accumulated gut damage in chronic infection could lower expected efficacy.

Liver function is important for the pharmacokinetics of BZD [64, 97], and reduced liver function could therefore interfere with anthelmintic activity. Infection with liver fluke, which causes liver damage in proportion to infection levels [62], and other liver diseases, could therefore affect BZD bioavailability and effectiveness, including against GIN. Reduced enzymatic activity of different liver microsomal function oxidases has been reported in sheep infected with *Fasciola hepatica* [29], also potentially altering patterns of xenobiotic metabolism and clearance of BZD anthelmintics [28]. The effects of other co-infections, and gut microbiome, on AE are unknown.

Many biocidal drugs, such as antimicrobials, require a competent immune system to achieve high efficacy. The extent to which immature immune systems impede AE in ruminants, as they do in mice [15], is unknown. Regardless of any age variation in actual AE due to immunity, the onset of immunity can confound the FECRT. In first-season grazing cattle, for instance, FEC typically peaks in the mid-part of the first grazing season and then declines due to immunity [73]. A FECRT conducted at this time could consequently over-estimate drug efficacy and yield a false-negative AR classification. The onset of immunity in lambs in Scotland concealed AR among *Teladorsagia circumcincta* for this reason [84]. Including an untreated control group in FECRT [16] allows non-therapeutic reduction in FEC to be accounted for, but is not always practical on farms [8]. Similarly, measurement of AE in sheep ewes during early lactation is tempting due to high FEC at this time [77], but is confounded by acute temporary loss of immunity during the peri-parturient rise and its rapid re-instatement.

## Parasite confounders

Different GIN species respond differently to anthelmintics through species-specific processes influencing bioavailability and lethal dose, irrespective of AR, and product licences and data sheets reflect these differences. The AE achieved against a mixed population of GIN in natural infections is therefore likely to vary with species composition, and such variation accentuated by differing levels of AR among species. Furthermore, the responses of parasite communities to treatment include physiological and evolutionary adaptations, and biotic interactions, which affect outcomes including FECR.

### Species composition

Calculation of overall FECR for GINs generally uses total FEC, separating only morphologically distinct taxa, e.g., moleinid (e.g., *Nematodirus*) versus trichostrongylid nematodes in ruminants. Concealed species variation, however, fundamentally affects FECR results. In mixed-species GIN infections in sheep in Scotland, FECR indicated AE of 65–77%, but *Teladorsagia circumcincta* dominated post-treatment FEC and was more highly resistant to both BZD and ML than undifferentiated FECR would suggest [59]. In cattle in Sudan, resistant *Haemonchus contortus* was found in cattle despite FECR exceeding 95% because of their low abundance relative to other species such as *H. placei* [68]. High levels of AR in minority species can therefore be missed by FECRT unless specific identification is included. The relative abundance of different GIN species varies widely between seasons and livestock age classes even within individual farms [25]. When AR is present to differing extents between GIN species, total FECR will also vary according to the proportions of different species present, as shown for mixed *Cooperia-Ostertagia* infection in cattle [38].

**Figure 2:**
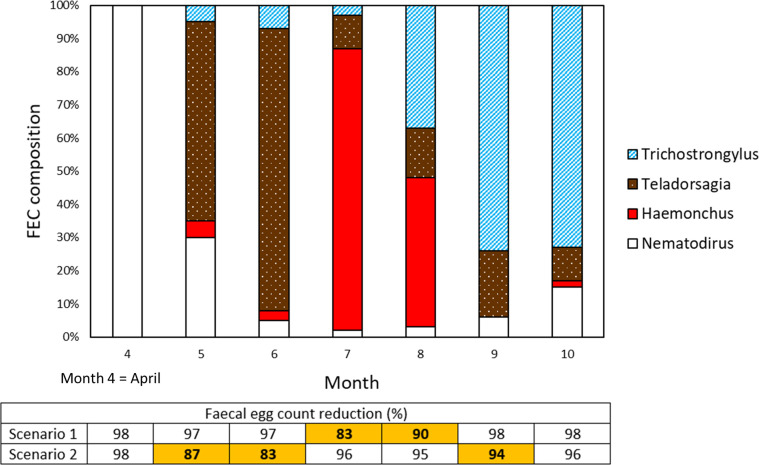
An illustrative example of the potential effect of seasonal shifts in nematode species composition on observed faecal egg count (FEC) reduction, based on typical epidemiological patterns in sheep in temperate areas. FEC composition indicates the proportion of eggs belonging to each species, where eggs of *Trichostrongylus* spp., *Teladorsagia circumcincta* and *Haemonchus contortus* are not easily distinguished from each other. Months are calendar months in the northern hemisphere, with *Nematodirus battus* and then *Teladorsagia* dominating in spring and early summer, *Trichostrongylus* in late summer and autumn, and *Haemonchus* transiently dominant following favourable climatic conditions [91]. In scenario 1, only *Haemonchus* is resistant to treatment, with FECR of 80%; in scenario 2, only *Teladorsagia* is resistant (80% FECR); FEC of other species reduce by 98% following treatment. A FECRT would have different results in different months, detecting resistance (<95% FECR) only in months (% FECR **in bold**) in which the resistant species contributes sufficiently to total faecal egg output, and returning false-negative results for AR in other months. The simulation does not account for differences in fecundity between species, which further amplify seasonal variation in FECR. Here, FECRT conducted at different times of year produce differing results even if anthelmintic efficacy is stable within species over that period.

Studies repeating FECR with species identification on individual farms over time are rare and difficult to achieve given requirements for adequate starting FEC levels [37], but seasonal variation in GIN species composition could clearly lead to misclassification of AR using undifferentiated FECRT (Fig. 2). Species identification is needed to resolve this difficulty, but is generally not available for AE or AR assessment on farms outside research studies. New molecular tools for rapid and accurate species identification may provide a viable path to widely available species-specific FECRT [5, 42]. Where species information is missing, however, AR classification based on FECRT effectively assumes that only a single species is present, or that efficacy is equal across species. This assumption is also implicit in simulations of the technical performance of FECRT [53, 54], therefore giving false confidence in the ability of FECRT to accurately classify the AR status of mixed populations.

### Non-linear responses to treatment

The egg output of different GIN species varies widely [18, 94, 95], such that the proportions of different species in mixed infections surviving treatment will not be accurately reflected in FEC: more fecund species will dominate FEC and bias FECR in their favour. Even within species, changes in the fecundity of surviving worms following treatment can confound FECR. Where worm fecundity is suppressed by density dependence, elimination of competing worms of the same or other species can release this constraint and lead to a temporary increase in egg output, masking worm mortality rate and potentially leading to false positive FECRT. In dogs, hookworms that survived pyrantel treatment produced more eggs, with FEC increasing by 41% in dogs with low-level resistant infections, despite removal of 71% of worms [41]. Density dependence also affects FECRT results in humans [43]. While its impact on the estimation of AE in livestock is not well-evidenced, density dependence in fecundity does occur in GIN of ruminants [9] and varies between parasite species [36].

Climatic conditions also affect fecundity, for example desiccation of *H. contortus* L3 decreases survival but increases subsequent fecundity [14]. Seasonal variation in both species composition [91] and life history traits within species has the potential to influence FECR and lead to natural variability in FECRT results, which are not due to measurement error. Interactions between helminth species, directly or by modifying host physiology, immunity or microbiome, also have the potential to influence effects of treatment on natural mixed infections in complex ways [17, 51]. If these act on short timescales, they might also affect FECR.

## Technical confounders

The stringent requirements of a formal FECRT for AR places it out of the practical reach of routine farm management, hence various more pragmatic approaches have been proposed to provide actionable information on AE against GIN. These, however, entail compromises that can affect the reliability of FECR under farm conditions. Statistical interpretation of the FECRT is not discussed in detail here. Although different statistical methods and the assumptions on which they are based affect the degree to which technical errors and other confounders alter AR classification, the underlying true FECR that they seek to interpret is also open to technical bias.

### Anthelmintic administration

Meticulous treatment is necessary for a defensible FECRT, including administration of the correct dose of anthelmintic for the weight of each individual animal included. When AE monitoring is aligned with normal farm management, however, such accuracy depends on having adequate facilities and sufficient time to weigh and dose individuals. A large proportion of farmers estimate anthelmintic dose by visual inspection or using the average weight of the group, risking under-dosing, while calibration of dosing equipment is infrequent [34]. Sub-therapeutic drug exposure due to incorrect administration or underestimation of body weight are likely causes of anthelmintic therapeutic failures in livestock, and might account for failure to confirm AR when rigorously repeating FECRT. For example, FECRT conducted by farmers or veterinarians found evidence of AR on 39% of 84 cattle farms in Belgium and Germany, but this was subsequently confirmed only on 25% [24]. The opportunities provided by anthelmintic treatments as part of normal farm management should therefore be taken to evaluate AE, but firm conclusions on AR require additional testing, e.g., using a formal FECRT. Due to dose variation inherent to topical formulations (see above), FECR following their use should not be used to evaluate AR.

### Sample collection, labelling, and storage

Pre-analytic factors such as collection, labelling, and storage of faecal samples prior to analysis can increase variability in FECRT results. Sampling faeces from the ground rather than *per rectum* is prone to allow egg development or contamination with free-living nematodes, while labelling errors can misattribute samples to treatment groups and cause serious misclassification. Storage of faecal samples can introduce inaccuracies through egg development or altered egg flotation after fixation [27, 70], hence refrigeration or vacuum packing are recommended if delayed laboratory analysis is unavoidable. Refrigeration, however, can affect subsequent egg development and species identification [20, 86], and hence compound inaccuracies in mixed species FECR as an indicator of AR.

### Sample size and faecal egg counting method

Aggregated distribution of helminths between individual animals in a group, and clumping of eggs within the faecal mass, require that adequate numbers of animals and faeces from each be included if FECR is to be representative of the group treated. The error structure of FEC and implications for the FECRT have been widely discussed and explored through simulation studies [8, 53, 54]. The impact of aggregated parasite distribution on FECR results can be lessened by ensuring that the same individuals are sampled before and after treatment, for example by recording ear tag numbers or applying temporary marks. Aggregation of eggs within faeces also makes it extremely important to take faecal samples that are large enough and thoroughly mix them before conducting a pooled or composite FEC [22, 31, 76]. Low sample weights, for example from young lambs or when sending material to external laboratories, might yield unrepresentative FEC.

The diagnostic portfolio for FEC includes firmly established methods, e.g. McMaster and Mini-FLOTAC [37], although results rely on operator proficiency. The availability of reliable, low-cost, easy-to-perform tools for swift FEC is of pivotal importance to increase user-friendliness and uptake of FECRT by veterinarians, advisors and farmers [66]. Different FEC methods apply different dilution factors to faeces, such that higher starting average FEC are needed to ensure robust FECRT results using less sensitive (= higher dilution) methods. Innovations in FECRT design include recommending that a minimum total number of eggs be counted [37], enabling statistically robust estimation of FECRT even at low starting densities. This will, however, tend to bias FECR towards the individuals shedding the highest numbers of eggs, increasing the vulnerability of results to suppressed AE due to individual state. For example, an individual animal in poor body condition, with reduced gut transit time, or under-dosed, could dominate average group-level FECR and lead to misclassification of AR. Such individuals should be carefully excluded from FECRT, but monitoring of AE is most practical when there is a need for treatment, which increases the likelihood of physiological conditions that impinge on AE. Statistical methods that consider the mean of individual FECR [7], rather than the reduction in group-average FEC, might be more robust to such individual physiological/pharmacokinetic variation.

### Climate and grazing

Increasingly variable weather conditions confound attempts to measure AR using FECRT, which require adequate starting egg density. In grazing livestock, delayed rise in GIN FEC in dry conditions will postpone the earliest date at which FECR might sensibly be measured; while follow-up of apparently low AE using a FECRT will also rely on adequate reinfection and hence weather and grazing conditions. Low observed FEC in summer could pressurize farmers and advisors to conduct FECRT at egg densities too low to produce accurate AR classification, or to defer the test to later in the year, increasing confounding by species composition and host immunity. Technical improvements in FEC methods that permit valid FECRT at lower starting FEC [37] could help to address these limitations.

## Implications of confounders for classification of resistance

The detection and management of AR is a major and growing challenge for sustained parasite management on farms, and hence for the sustainability of grass-based livestock production. Confirmation of AR requires *post mortem* worm counts in animals following treatment, which is rarely feasible on farms, leaving the FECRT as the only appropriate test for AR that is widely applicable between GIN species and anthelmintic classes. The failure of the livestock industry to adequately monitor anthelmintic efficacy has much to do with the limitations of this test, which is onerous and expensive if done thoroughly, and often leaves considerable uncertainty around the results of a particular test and its repeatability at farm level. Simplification of the FECRT in an effort to align its application with routine parasite treatments and so increase uptake, however, adds to variability in results due to the real-world confounders discussed here. The same dilemma applies to epidemiological studies of AR, which must trade sample size against effort per farm, limiting the strength of conclusions that apply rigorous FECRT but on a small number of farms [26, 78].

Although the factors listed above are highly likely to be common on farms and hence to influence FECR results, the extent to which they actually confound estimates of AE and AR is not well known. There are good reasons for this. Following up therapeutic failure with formal FECRT requires resources, commitment and competent advisors, and is not always feasible. The achievement of high efficacy in drug licensing trials despite natural variation in animal and parasite physiology might also suggest that many of the factors listed are only potential confounders, whose impact in reality is not large enough to materially affect FECR results and AR classification. As AR takes hold, however, efficacy becomes increasingly marginal and confounders become more important to outcomes. Furthermore, therapeutic failure will accentuate the impact of parasites on the health and physiology of livestock and reinforce parasite-dependent confounders. As AR takes hold and testing rates remain low [78], the situation of evaluating anthelmintic efficacy only following failure of previous attempts at control is likely to become more common [92], and these confounders consequently more important. Increased unpredictability in the epidemiology of the major helminth parasites of livestock will also inflate impacts of confounders, such as seasonal variation in species composition.

While hard evidence for the role of confounders in AE and AR classification is hard to come by, there are strong grounds to be vigilant. Refinements such as improved FEC methods and statistical interpretation of FECRT results are helpful, but they do not address the full range of potential confounders of FECR results. It is important that veterinary professionals and other advisors, as well as parasitologists and animal scientists, are aware of these, and that expectations of the FECRT as a standard test are appropriately managed, especially when modified to improve practicality. False expectations of a clear and lasting result from a single FECRT can lead to inappropriate management decisions, which have important implications for sustained parasite control, and can be avoided if potential confounders of test results are appreciated. Ultimately, a wider array of approaches to monitoring AE on farms is needed to more fully track trajectories and to adjust parasite management strategies in an evidence-based and timely manner.
